# Feasibility of Postmortem Imaging Assessment of Brain: Liver Volume Ratios with Pathological Validation

**DOI:** 10.1159/000497158

**Published:** 2019-04-10

**Authors:** Susan C. Shelmerdine, Kimberly L. Chung, John C. Hutchinson, Claire Elliott, Neil J. Sebire, Owen J. Arthurs

**Affiliations:** _a_Department of Radiology, Great Ormond Street Hospital for Children NHS Foundation Trust, London, United Kingdom; _b_Department of Histopathology, Great Ormond Street Hospital for Children NHS Foundation Trust, London, United Kingdom; _c_Developmental Biology and Cancer Programme, UCL Great Ormond Street Institute of Child Health, London, United Kingdom; _d_Imaging and Biophysics, UCL Great Ormond Street Institute of Child Health, London, United Kingdom

**Keywords:** Postmortem, Radiology, Fetal growth restriction, Magnetic resonance imaging, Organ volumes, Brain, Liver ratio

## Abstract

**Introduction:**

Organ volumes at postmortem magnetic resonance imaging (PMMR) should reflect autopsy organ weights, and thus brain: liver volume ratios on imaging could be a surrogate for weight volume ratios at autopsy to indicate fetal growth restriction (FGR). This study aims to determine whether imaging-based organ volume ratios can replace autopsy organ weight ratios.

**Materials and Methods:**

An unselected cohort of perinatal deaths underwent PMMR prior to autopsy. Semiautomated brain and liver volumes were compared to autopsy organ weights and ratios. Ratios were compared using Bland-Altman plots, and intra- and interobserver variability was assessed.

**Results:**

A total 49 fetuses (25 male, 51%) at 17–42 weeks gestation were ­assessed. There was a reasonable correlation between autopsy-derived brain: liver weight ratios (AB: LwR) and imaging-derived brain: liver volume ratios (IB: LvR; *r* = 0.8). The mean difference between AB: LwR and IB: LvR was +0.7 (95% limits of agreement range −1.5 to +2.9). In a small subset where FGR was present, the optimal IB: LvR ≥5.5 gave 83.3% sensitivity and 86.0% specificity for diagnosis. There was acceptable agreement within readers (mean difference in IB: LvRs 0.77 ± 2.21) and between readers −0.36 ± 0.68.

**Conclusion:**

IB: LvR provides a surrogate evaluation of AB: LwRs, and may be used as a marker of FGR where autopsy is declined.

## Introduction

Fetal growth restriction (FGR) is the single largest contributing factor to perinatal mortality in anatomically normal fetuses [1]. Although sometimes used interchangeably with small for gestational age (SGA), FGR consists of specific features of malnutrition and in utero growth impairment irrespective of the growth percentile [2]. While FGR contributes significantly to fetal morbidity and mortality, it is also associated with an increased recurrence risk for future pregnancies likely through placental mechanisms. Accurate diagnosis of FGR is therefore important in antenatal and postmortem investigations.

FGR may be assessed at autopsy, where a potentially important feature is the result of blood diversion to spare the growth of brain and heart at the expense of other organs such as the liver [3]. Several studies have indicated that the ratio of brain to liver weights at autopsy can indicate FGR. It was initially suggested that autopsy-derived brain: liver weight ratios (AB: LwR) > 3 may indicate a fetus with an elevated risk of FGR [4], but a large autopsy study demonstrated that a ratio > 5 or 6 is required, with superimposed effects of maceration following intrauterine demise [5].

Despite its recognized benefits, acceptance rates of pediatric autopsy are low (12% in the USA, 15% in the UK [6]). In recent decades, postmortem imaging has increasingly been used and accepted as an adjunct with autopsies or as an alternative when parental consent is refused [7]. Postmortem magnetic resonance imaging (PMMR) has been shown to have a high diagnostic accuracy and parental acceptability when compared to autopsy [8, 9] and 3D organ volumes on PMMR reflect organ weights at autopsy with a high level of accuracy [10, 11, 12]. This may negate the need for invasive autopsy for organ weight measurements. Small studies using antenatal MRI have also shown that fetal imaging-derived brain: liver volume ratios (IB: LvR) may be a marker of FGR in late-gestation live fetuses [13, 14], and thus postmortem MRI may be able to provide these data [15]. In this study, we investigate the feasibility of whether PMMR IB: LvRs could be a surrogate marker for weight ratios by comparing imaging data to traditional autopsy organ weight measurements.

## Materials and Methods

### Patient Selection

A 5-year retrospective review of our local pathology database from July 2012 to July 2017 was conducted for all fetuses that had undergone both PMMR and autopsy. Cases were included where brain and liver weight measurements at autopsy were available. Cases were excluded where imaging was non-diagnostic, or an abnormality was found in the brain or liver on PMMR or subsequent autopsy.

In cases where there was a diagnosis of FGR, this was defined on antenatal ultrasound examination as estimated fetal weight below the 5th centile in the presence of ultrasound Doppler abnormalities. All autopsies were performed for patients by 1 of 4 consultant pathologists at our institution according to national protocols. Organ weights were performed on an electronic scale as part of the conventional autopsy procedure. The times from death to imaging and death to autopsy were also recorded.

Written informed consent was obtained from all parents of participants for clinical preautopsy PMMR, as part of our institution's postmortem imaging protocol. This study was performed as part of an ethically approved larger study investigating minimally invasive autopsy techniques and novel methods of postmortem imaging (CE13/LO/1494 and CE2015/81).

### Imaging Protocol

Consent was obtained in all cases for imaging from the patients' parents. All cases were kept in the mortuary at 4°C prior to PMMR. The imaging was performed prior to autopsy on a 1.5-T MRI scanner for all cases (Avanto, Siemens Medical Systems, Erlangen, Germany) using whole body three-dimensional (3D) T2-weighted turbo spin echo (TR 3500 ms, TE 27 ms, voxel size 0.8 mm^3^, 2 averages), 3D T1-weighted volumetric interpolated breath-hold examination (TR 5.9 ms, TE 2.4 ms, flip angle 25°, voxel size 0.8 mm^3^, 8 averages) and 3D constructive interference in the steady state sequence (TR 9.2 ms, TE 4.6 ms, flip angle 70°, voxel size 0.6 mm^3^, 4 averages), as published previously in our protocol [16].

### MRI-Based Volumetry

**Fig. 1:**
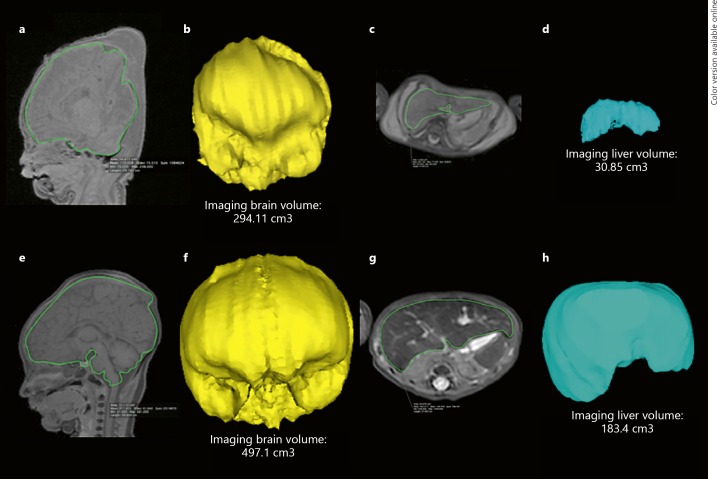
PMMR of a stillborn 39-week gestational age fetus with FGR (**a–d**) and a stillborn 40-week non-FGR (control) patient (**e–h**). MRI manual segmentation on isovolumetric T1-weighted sequences of the brain are shown in sagittal views (**a**, **e**, green outline) with corresponding derived 3D volumes (**b**, **f**). A similar method was adopted on isovolumetric T2-weighted imaging of the abdomen for the liver in axial sections (**c**, **g**, green outline) with corresponding derived 3D volumes (**d**, **h**). For the fetus with FGR (**a–d**), the IB: LvR was calculated as 9.53 (AB: LwR was 7.6), and for the control fetus without FGR (**e–h**) the IB: LvR was 2.7 (AB: LwR was 2.4).

Organ volumes were calculated using Osirix MD v.2.9 64 (Pixmeo SARL, Bernex, Switzerland) for Macintosh. This was conducted manually by outlining the contours of the brain and the liver on the 2D image data from the isovolumetric T1 sequences (for the brain) or T2-weighted sequences (for the liver) on each image slice to generate a volume measurement (cm^3^). Both brain and liver volume measurements were repeated in two different planes − coronal and sagittal for brain imaging, axial and coronal for liver imaging (Fig. 1).

For brain volume estimation, the inferior border was defined as the foramen magnum. Extra-axial fluid and venous sinuses were excluded where possible; however, the pituitary gland was included. The volume estimation was not conducted in the axial plane given the difficulty in excluding the basal skull bones from the underlying brain parenchyma. For liver volume estimation, the main portal vein, hepatic trifurcation, and gallbladder were excluded where possible.

### Measurements

All PMMR organ volumes were conducted by a single observer (K.L.C.) blinded to the patient's demographics and antenatal history. Intraobserver variability was examined by the same observer repeating brain and liver organ volume measurements (in two planes) for a random set of 10 cases, with a 1-week time interval. Interobserver variability was examined by a second blinded observer (S.C.S.) measuring 5 different randomly selected cases in both planes.

### Statistical Analysis

Individual IB: LvRs were then calculated for all cases, and compared to the autopsy brain: liver weight ratios. The difference in ratios was evaluated by Bland-Altman differences of mean values and Pearson's correlation test. Bland-Altman plots were also used to assess inter- and intraobserver variability. All measurements were calculated using SPSS.

## Results

We identified 60 unselected fetuses that underwent PMMR and complete autopsy with organ weights. We excluded 11 cases for intracranial pathology (ventriculomegaly or tumor, *n* = 5), non-diagnostic PMMR for organ volumetry (*n* = 2), and incomplete antenatal history (*n* = 4). A total 49 fetuses (25 male, 51%) ranging between 17 and 42 weeks gestation were included in the final cohort. Eight (16.3%) were neonatal deaths (0–15 days old at death, mean 5 days, 25–40 weeks gestation, mean 32 weeks) and 41 (83.7%) were stillborn (17–42 weeks gestation, mean 29 weeks). The demographic and imaging details of the cases are presented in Table 1.

There were 4 cases with a clear clinical history of FGR: in 2 of these patients the placenta demonstrated fetal vascular malperfusion at autopsy, and in 2 patients the placental pathology was non-contributory. There were 45 cases without any documented history of growth retardation or significant placental pathology.

### Overall Correlation between Imaging Organ Volumes and Autopsy Weights

We found that sagittal brain volume measurements correlated better than coronal volumes to brain weights at autopsy (*r* = 0.961 vs. 0.958, respectively). Similarly, we found that axial liver volumes correlated better than coronal volumes (*r* = 0.984 vs. 0.969, respectively) to liver weights at autopsy. Thus, we chose to use sagittal brain and axial liver volume measurements for the most accurate volume representation of brain/liver weight ratio.

**Fig. 2:**
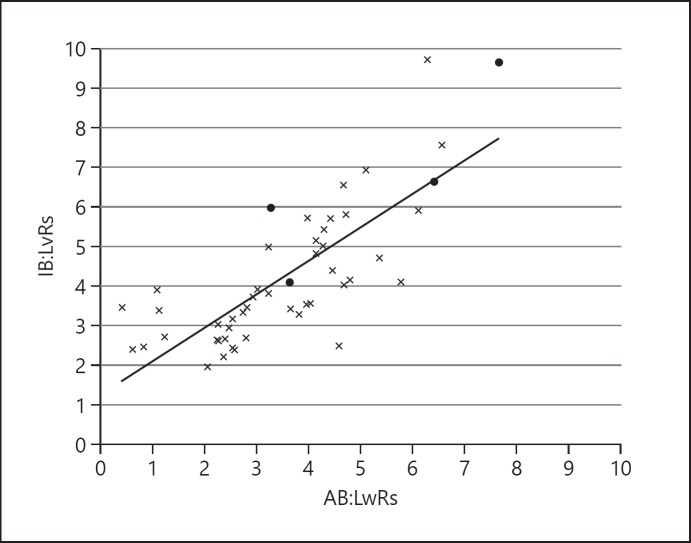
The overall correlation between IB: LvRs and AB: LwRs was reasonable with a correlation coefficient of *r* = 0.8. Control cases are denoted by crosses (×, *n* = 45) and suspected FGR cases by black circles (⚫, *n* = 4).

**Fig. 3:**
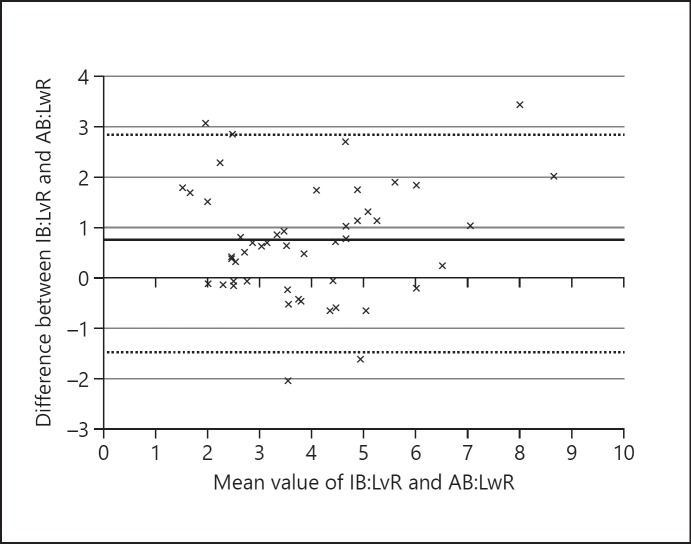
Bland-Altman plot showing that mean differences and 95% LOA between IB: LvR and AB: LwR. The mean difference is represented by a solid black line (+0.7), the 95% LOA are denoted by dashed lines (ranging from −1.5 to +2.9).

The mean AB: LwR was 3.6 ± 1.64 (range 0.4–7.6) and mean IB: LvR was 4.3 ± 1.8 (range 2.0–9.7). There was a reasonable correlation between AB: LwR and IB: LvR, with a correlation coefficient of *r* = 0.8 (Fig. 2). The mean differences between AB: LwR and IB: LvR was +0.7 (95% limits of agreement, LOA, ranging from −1.5 to +2.9; Fig. 3). The average absolute difference in measurements was 1.0 ± 0.9. The average of differences in values between IB: LvR and AB: LwR was 0.7 ± 1.1 (LOA −1.5 to 2.2).

### Determining FGR

**Fig. 4:**
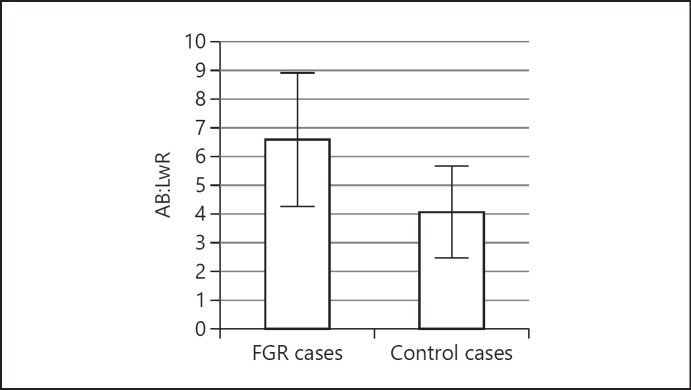
Bar chart demonstrating the mean AB: LwR and 95% CIs between FGR cases and control “non-FGR” cases. The mean AB: LwR was significantly higher in FGR fetuses (FGR 6.58 ± 2.31, *n* = 4, vs. normal 4.07 ± 2.31, *n* = 45, *p* < 0.01).

In a small subset of our patients (*n* = 4) there was an antenatal history of FGR. There was an acceptable correlation between IB: LvR and AB: LwR for both FGR (*r* = 0.9) and normal fetuses (*r* = 0.7). The mean AB: LwRs were significantly higher in FGR fetuses than normal fetuses (FGR 6.58 ± 2.31, *n* = 4, vs. normal 4.07 ± 2.31, *n* = 45, *p* < 0.01; Fig. 4). In order to determine the optimal cut-off IB: LvR value for potentially diagnosing FGR, we calculated the diagnostic accuracy rates for different IB: LvRs compared to AB: LwR (Table 2). We found that an IB: LvR ≥5.5 gave the best combination of high sensitivity, specificity, and negative predictive value, although our case numbers are small and thus confidence intervals are wide.

### Effect of Maceration

The effect of maceration on organ volume and weight is given in Table 3. The mean brain volume and weights appear to increase with increasing maceration, although this was not found to be statistically significant (*p* = 0.24 and 0.15, respectively), whereas the mean liver volumes and weight decrease significantly with increasing maceration (*p* < 0.05); thus, maceration has a significant confounding effect on brain: liver volume and organ ratio, exaggerating these ratios.

### Agreement

There was acceptable intrareader agreement with mean difference in IB: LvRs of 0.77 ± 2.21 (95% LOA −1.44 to 2.97), brain volumes of 8.42 ± 14.91 cm^3^ (95% LOA −20.81 to 37.65), and liver volumes of −3.32 ± 3.47 cm^3^ (95% LOA −10.12 to 3.48). The average IB: LvR difference in measurements between readers was −0.36 ± 0.68 (95% LOA −0.32 to 1.04), with brain volumes of −7.29 ± 6.93 cm^3^ (95% LOA −20.87 to 6.29) and liver volumes of −0.31 ± 0.39 cm^3^ (95% LOA −1.08 to 0.46).

## Discussion

This study shows the feasibility of performing organ volumes at PMMR and confirms that they reflect organ weights at autopsy. This represents a significant advance for non-invasive autopsy methods, as organ brain: liver volume ratios at postmortem imaging could represent a potential surrogate measurement. Although our population cohort contained a small number of cases of FGR, we showed that an IB: LvR > 5.5 could be helpful in identifying FGR fetuses, in keeping with data derived from a larger series [5]. Maceration is a confounding variable in the postmortem setting, but even in this context these results may still be useful for parents who decline invasive autopsy in the future.

Our study is consistent with a detailed study of over 1,000 intrauterine deaths [5] which found that there was a significant difference in the AB: LwR between SGA cases with placental histological abnormalities (FGR; median ratio 6) and other SGA cases (median ratio 3). Organ volumes have been reliably found to represent organ weights at different PMMR field strengths [10, 11, 12], but none of these studies have demonstrated differences in brain: liver volume between groups. A small study using antenatal MRI has shown that IB: LvRs may be a marker of FGR in late gestation live fetuses [14], but our study is the largest case series where this measurement has been applied in the perinatal postmortem setting.

AB: LwR has traditionally been used to indicate increased risk of FGR and perinatal morbidity, although we also found an overlap between B: LvR and AB: LwR measures between normal and FGR fetuses. The causes of FGR are diverse, and not all types of FGR may result in an increased brain: liver ratio [17]. More recently, FGR has been classified into three categories: type 1, symmetrical FGR with decreased growth in early pregnancy; type 2, asymmetrical FGR which manifests typically after 30–32 weeks gestation and is primarily attributed to reduced cell size, and type 3, mixed FGR with a combination of reduced cell proliferation and growth as seen in types 1 and 2. While the brain: liver ratio may be suitable for indicating certain asymmetrical FGR, it may be inappropriate in symmetrical FGR, but this would apply equally to both traditional autopsies and PMMR.

Even if we accept that IB: LvR estimation using PMMR is a reliable surrogate of this measure, the precise cut-off ratio to determine FGR is unknown. In conventional pathological autopsy findings, Mitchell et al. [4] found that among 182 stillbirths, those with SGA have the same proportion of fetuses above and below an AB: LwR ratio of 3. The same study found a second cluster of SGA cases with an AB: LwR of 8–12, which may be class 1 FGR, but with extensive overlap. Damodadam et al. [13] found that IB: LvR above 3 is associated with 3.3-fold increase in growth restriction and perinatal mortality through in utero MRI imaging in late gestation fetuses. Stephens et al. [17] reported that an AB: LwR above 3 for fetuses over 28 weeks gestation and 3.7 for more preterm babies will detect FGR. AB: LwR > 5 can better predict FGR when cases that are chromosomal abnormalities due to a congenital central nervous system are excluded. Breeze et al. [15] found the IB: LvR estimated at postmortem MRI imaging among 25 fetuses of gestational age between 16 and 40 weeks suggested a cut-off of 4.1 achieved 45% sensitivity and 100% specificity for FGR. Lastly, Man et al. [5] found that an AB: LwR of 6 differentiated placental FGR from SGA without significant placental pathology, with a sensitivity of 53% and specificity of 80%, although there was no AB: LwR that absolutely distinguished these groups. Our study found that an IB: LvR > 5.5 gave the best overall highest sensitivity, specificity, and negative predictive value, although there was still an overlap between normal and abnormal cases (Fig. 1). For this reason, whilst all IB: LvR cut-offs ≥4 give adequate sensitivity, our sample size is small and confidence intervals are relatively wide, and thus a higher IB: LvR ≥5.5 would be more conservative and lead to a more accurate detection of FGR cases.

Different postmortem changes can affect the interpretation of postmortem imaging, in particular maceration [18]. Maceration is known to alter internal organ weights, but is proposed to be largely due to fluid retention [19]. Our findings match those of previous studies whereby liver weight is more severely affected than brain weight by maceration severity. Our results on the effect of maceration on individual organs match their observed changes in AB: LwR from 2.6 in non-macerated fetuses to 4.5 in severely macerated fetuses [19], with similar changes in our IB: LvR (Table 3). Organ weight loss or gain clearly affects both AB: LwR and IB: LvR, and thus needs to be taken into account when estimating FGR in macerated fetuses [17]. One limitation in our study was the lack of available antenatal notes to compare fetal biometry with postmortem findings in order to quantify the amount of organ maceration. Nevertheless, our imaging results alone correlate well with autopsy findings.

The largest limitation, however, is the number of available cases that underwent PMMR and a complete autopsy with complete antenatal records to distinguish FGR from normal fetuses. Only 4 cases met our criteria of FGR within our retrospective cohort. Nevertheless, our IB: LvRs were comparable to AB: LwRs from autopsy and feasible to measure, which was the main aim of this study. More reliable cut-offs may be determined with a larger cohort. Imaging is also limited by gestational age or size variation: earlier gestation fetuses gave lower resolution PMMR imaging which may result in non-diagnostic imaging [20] or difficulty in delineating regions of interest [18].

## Conclusion

In conclusion, this study has shown that PMMR-derived IB: LvR reflects AB: LwR in a small study cohort, with a significant difference in IB: LvR between normal and FGR fetuses in a small sample. Organ volumes from postmortem imaging are an alternative method of providing organ data for parents who decline conventional autopsy, even in the setting of suspected FGR. This could provide a non-invasive method for identification of FGR following perinatal death. The value of postmortem imaging alongside antenatally determined factors and placental evaluation should now be assessed in a larger cohort.

## Statement of Ethics

Written informed consent was obtained from all parents of participants for clinical preautopsy PMMR, as part of our institution's postmortem imaging protocol.

This study was performed as part of an ethically approved larger study investigating minimally invasive autopsy techniques and novel methods of postmortem imaging. The ethical approval was provided by the National Research Ethics Service Committee, UK (REC references: CE13/LO/1494 and CE2015/81).

## Disclosure Statement

The authors have no conflicts of interest to declare.

## Funding Sources

S.C.S. is supported by a RCUK/UKRI Innovation Fellowship and Medical Research Council (MRC) Clinical Research Training Fellowship (grant ref. MR/R00218/1). This award is jointly funded by the Royal College of Radiologists (RCR). O.J.A. is funded by a National Institute for Health Research (NIHR) Clinician Scientist Fellowship (NIHR-CS-012–002), and N.J.S. is funded by an NIHR Senior Investigator award. The authors receive funding from the Great Ormond Street Children's Charity and the Great Ormond Street Hospital NIHR Biomedical Research Centre. This article presents independent research funded by the MRC, RCR, NIHR, and the views expressed are those of the author(s) and not necessarily those of the NHS, MRC, RCR, the NIHR, or the Department of Health.

## Author Contributions

All authors have made substantial contributions to the study and endorsed the data and conclusions. K.L.C., S.C.S., and C.E. performed data collection, imaging interpretation, and data analysis. Study conception and design was by O.J.A. N.J.S. and O.J.A. contributed towards the interpretation of results, data analysis, and critical appraisal of the report. All authors contributed toward writing the paper.

## Figures and Tables

**Table 1:** Summary of the demographics and sample size of our study population

	Overall (*n* = 49)	FGR group (*n* = 4)	Control group (*n* = 45)
Male	25 (51)	3 (75)	22 (49)^ns^
Gestational age, weeks	30±7.4 (17–42)	33±7.5 (22–39)	29±7.4 (17–42)^ns^
Age at death, days	0±3.5 (o–19)	0±0 (0–0)	1±3.7 (0–19)^ns^
Maceration score	1±1.2 (0–3)	1±1.3 (0–3)	2±1.2 (0–3)^ns^
PM imaging interval (delivery to imaging), days	8±3.2 (2–14)	6±2.4 (3–8)	8±3.2 (2–14)^ns^
Autopsy interval (delivery to autopsy), days	10±5.1 (2–29)	8±1.6 (6–10)	10±5.2 (2–29)^ns^
Time from imaging to autopsy, days	3±3.9 (0–24)	2±2.2 (0–5)	3±4.1 (0–24)^ns^
Mode of death			
Miscarriage	4 (8)	0	4 (9)*
Termination of pregnancy	19 (39)	1 (25)	18 (40)^ns^
Stillbirth/intrauterine death	18 (37)	2 (50)	16 (36)^ns^
Neonatal death	8 (16)	1 (25)	7 (15)^ns^

Data are presented as *n* (%) or the mean ± 95% confidence intervals (range).

* *p* < 0.05, statistically significant difference. FGR, fetal growth restriction; ns, not statistically significant.

**Table 2:** Predictive effect of increasing IB: LvRs as cut-off values for FGR

IB: LvR cut-off	TP/FP, *n*	FN/TN, *n*	Sensitivity (95% CI)	Specificity (95% CI)	PPV (95% CI)	NPV (95% CI)	Concordance (95% CI)
≥4	19/3	3/24	86.4 (66.7–95.3)	88.9 (71.9–96.1)	86.4 (66.7–95.3)	88.9 (71.9–96.1)	87.8 (75.8–94.3)
≥4.5	9/8	5/27	64.3 (38.8–83.7)	77.1 (61.0–87.9)	52.9 (31.0–73.8)	84.4 (68.2–93.1)	73.5 (59.7–83.8)
≥5	6/9	2/32	75.0 (40.9–92.9)	78.0 (63.3–88.0)	40.0 (19.8–64.3)	94.1 (80.9–98.4)	77.6 (64.1–87.0)
≥5.5	5/6	1/37	83.3 (43.6–97.0)	86.0 (72.7–93.4)	45.5 (21.3–72.0)	97.4 (86.5–99.5)	85.7 (73.3–92.9)
≥6	4/3	1/41	80.0 (37.6–96.4)	93.2 (81.8–97.7)	57.1 (25.0–84.2)	97.6 (87.7–99.6)	91.8 (80.8–96.8)

TP, true positive; FP, false positive; FN, false negative; TN, true negative; PPV, positive predictive value; NPV, negative predictive value.

**Table 3:** Effect of maceration on organ volumes, weights, IB: LvRs, and AB: LwRs

Maceration score	Sample size, *n*	Brain volume, cm^3^	Brain weight, g	Liver weight, g	Liver volume, cm^3^	Mean IB: LvR	Mean AB: LwR
0	16	173.8 (156.8) [35.4–497.1]	173.6 (163.0) [23.0–493.9]	60.9 (54.1) [7.0–205.7]	56.1 (49.7) [7.3–183.8]	3.24 (1.0) [1.96–5.97]	2.99 (1.0) [0.82–4.78]

1	13	179.0 (152.9) [21.3–420.3]	166.6 (164.7) [12.8–425.8]	61.6 (60.2) [3.8–185.7]	51.0 (49.0) [3.7–158.8]	3.86 (1.3) [2.41–6.64]	2.82 (1.8) [0.63–6.40]

2	8	235.0 (141.7) (51.9–425.2)	232.5 (148.9) [9.1–420.0]	62.9 (45.4) [15.9–143.0]	48.5 (36.3) [14.6–112.9]	5.19 (2.2) [3.48–9.71]	3.76 (1.8) [0.42–6.27]

3	12	218.4 (121.2) (51.2–400.8)	255.4 (136.6) [42.4–435.0]	50.6 (27.4) [12.8–92.0]	40.2 (21.2) [10.2–70.6]	5.52 (1.9) [2.53–9.65]	5.02 (1.2) [3.24–7.65]

Data are presented as the mean (SD) [range]. The maceration scores were obtained via a subjective scoring by the consultant pathologist at autopsy ranging from 0 (no maceration) to 3 (significant maceration).
